# A Comparison of the Modified Score for the Assessment of Chronic Rheumatoid Affections of the Hands and the Australian/Canadian Osteoarthritis Hand Index in Hand Osteoarthritis Patients

**DOI:** 10.1155/2009/249096

**Published:** 2009-02-10

**Authors:** Judith Sautner, Ingrid Andel, Bernhard Rintelen, Burkhard F. Leeb

**Affiliations:** ^1^Lower Austrian Centre for Rheumatology, 2nd Department of Medicine, State Hospital Weinviertel Stockerau, 2000 Stockerau, Austria; ^2^Karl Landsteiner Institute for Clinical Rheumatology, 2000 Stockerau, Austria

## Abstract

*Objectives*. To compare the modified score for the assessment and quantification of chronic rheumatoid affections of the hands (M-SACRAH) with the Australian/Canadian osteoarthritis hand index (AUSCAN) in hand osteoarthritis (HOA). Both are self-administered patient questionnaires, being designed to assess functional status, stiffness, and pain in affected patients, despite some differences in format, compass and arrangement of questions. *Methods*. 66 HOA patients (51 females), attending the outpatient clinic, were included. Patients completed the AUSCAN (15 visual analogue scales) (VAS) and the M-SACRAH (12 VAS). *Results*. AUSCAN-pain amounted to a mean of 41.9 (±2.9 SEM), AUSCAN-stiffness to 53.1 (3.7) and AUSCAN function to 42.6 (3.2). M-SACRAH-function amounted to 25.4 (2.4), M-SACRAH-stiffness to 42.6 (3.0), and M-SACRAH-pain to 43.7 (3.1). The total mean M-SACRAH was 37.2 (2.4) (all *P*'s < .0001). The three respective domains of the two scores correlated significantly: pain: *r* = 0.73, stiffness: *r* = 0.75, and function: *r* = 0.76 (all *P*'s < .0001). The four identical items in both scores also correlated significantly. No significant gender specific differences were observed. *Conclusion*. Despite a different scope of items, a significant high correlation of these two scores evaluating HOA patients could be demonstrated. We conclude that both scores are equivalently valuable for the assessment of health status in these patients.

## 1. Introduction

Hand osteoarthritis (HOA) is a highly prevalent condition, which can result in considerable disability. [1] In comparison with the diagnostic and therapeutic efforts concerning knee or hip OA, HOA was neglected for quite some time—either due to its unspectacularity or due to a number of difficulties in its diagnosis and classification. Driven by European Standing Committee for International Clinical Studies including Therapeutics (ESCISIT), separate evidence-based recommendations for hip and knee OA were developed between 2000 and 2005. In September 2005, the members of the EULAR OA task force met for the first time to start developing recommendations primarily for the management and afterwards for the diagnosis of HOA—thus emphasizing the importance of this clinical topic [2, 3].

Already in 1999, the Osteoarthritis Research Society International (OARSI) agreed upon core outcomes for clinical trials in hand osteoarthritis (HOA) and these efforts have been renewed at the OARSI OA meeting in Boston in December 2005 [4, 5]. These respective core outcomes include pain, functional index, patient's global assessment (PGA), structure, number of painful or tender joints, grip strength, and pinch strength. There was further agreement that patients' self-reported difficulty with daily activities should be assessed with a valid and reliable measure [4].

Some efforts to develop scoring systems for rheumatoid affections of the little finger joints have been made in the past. Two of them, the algofunctional index functional index for hand osteoarthritis (FIHOA) by Dreiser and the rheumatoid hand functional disability scale by Duruoz only assess the functional handicap of patients [6, 7]. These two scores, furthermore, are administered by interviewers which might result in bias due to interaction between the patient and the interviewer [8].

We attempted to create a self-administered instrument which did not only include a functional index but also incorporated pain and stiffness and therefore designed the score for the assessment and quantification of chronic rheumatic affections of the hands, the SACRAH, in 1999 [9].

Bellamy et al. created a comparable score, following the well-established pattern of the WOMAC and exclusively dealing with HOA, the AUSCAN [8, 10, 11]. In this context, it seems questionable, whether an instrument is capable of targeting HOA exclusively.

As both AUSCAN and M-SACRAH meet the requirements of the OARSI hand osteoarthritis core outcomes, yet have been designed by different approaches, we compared both scores in one HOA patient group to verify possible differences or similarities in the assessment of the status and outcomes of these patients. Special emphasis of our investigation was put on possible differences between the two scores concerning their results in different genders. A further goal was to investigate possible differences in the responsiveness of the two scores to therapeutic interventions.

## 2. Material and Methods

Between August 2003 and April 2005, the four authors were assessing patients at the outpatient clinic of our department. Having been diagnosed with HOA according to the ACR criteria by one of the four, patients were included consecutively into the study [12]. Thus, sixty six outpatients completed both questionnaires.

### 2.1. Questionnaires under Comparison

#### 2.1.1. SACRAH/M-SACRAH

In brief, the SACRAH encompasses 23 VAS (100 mm) covering the three categories functional impairment (18 items), stiffness (2 items), and pain (4 items). This score has been validated in 69 HOA and 103 RA patients [13].

In 2004, we presented a modified and shortened version of the SACRAH, the M-SACRAH to simplify the questionnaire's use for the patient [14].

The M-SACRAH—as well as its predecessor, the SACRAH—has been established and validated in German and also consists of 3 domains including 12 items, namely, 8 targeting function, 2 targeting stiffness, and 2 targeting pain. It has, meanwhile, been translated to English according to standardized procedures. Meanwhile, the questionnaire has been translated to English and Serbocroatian according to standardized procedures and has been validated in a Serbian patient group [15]. Data of the principal component analysis are given in Table 6.

#### 2.1.2. AUSCAN-Questionnaire

The AUSCAN has been established in English, exclusively dealing with HOA patients and finally published in 2002. It exists in a Likert version, a numerical rating scale (NRS), and in a VAS format [8, 10, 11]. It comprises 15 items covering pain (*n* = 5), stiffness (*n* = 1), and function (*n* = 9). The distribution of questions among the three categories, however, is to some extent different from the M-SACRAH.

For the purpose of this study, the VAS-version was used, as the M-SACRAH also encompasses VAS-scales. As the AUSCAN questionnaire is not in the public domain and thus not unrestrictedly available, the candidate items from the publication were used to generate a German version for this specific study. This was done by two professional English-German translaters, one translating the questionnnaire from English into German, the other one translating it back into English. Since the English translation from the German version matched the original AUSCAN questionnaire, the German version was considered primarily valid for the use in this study. The detailed items of the questionnaire are shown in Table 1. Construct validity of this German version was assessed carrying out principal component analysis (Table 7).

The main difference between the two questionnaires relates to the importance of stiffness and pain. While the AUSCAN distinguishes between pain on several activities, the M-SACRAH asks for pain at work in general and for pain at rest. Regarding stiffness only morning stiffness is covered by the AUSCAN. The M-SACRAH, however, also asks for starting stiffness during the day.

#### 2.1.3. Completion of the Questionnaires and Further Assessments

After initial instruction by a nurse or a resident, as to how the questionnaires should be tackled, the participants completed both forms without further assistance in random order, one right after arrival and the other one just before the assessment by the physician, resulting in a mean time lag of half an hour. All questionnaires were completed during outpatient department hours between 9 a.m. and 1 p.m. Patients thereafter underwent a clinical examination with an assessment of their complete joint status, also including patient's global assessment (PGA)and physician's global assessment (PhGA) (100 mm VAS).

PGA was assessed by the treating physician, using the phrase “Please indicate how severe you are compromised by your hand osteoarthritis during the last 48 hours!”

### 2.2. Statistical Methods

Statistical evaluation was carried out using SPSS 11.0 for Windows. As all the relevant parameters proved to be normally distributed according to Kolmogorov-Smirnoff accomodation, parametric tests were applied. Results are presented as mean (±standard error of the mean = SEM) for continuous variables. Correlations of continuous variables were performed using Pearson's correlation. Groups were compared using the Student's *t*-test. *P*-values <.05 were considered statistically significant.

In order to evalutate dimensionality and factorial structure of both scores, and to reveal whether scale items eventually cross-load on more than one factor, exploratory factor analysis by principial component analysis (PCA) was performed (Tables 5 and 6).

Factor analysis, including variations such as PCA, is a statistical approach which is applied to analyze interrelationships among a large number of variables and to explain these variables with respect to their common underlying dimensions (factors). The objective is the attempt to condense information contained in a number of original variables into a smaller set of variates (factors) with a minimum loss of information [16]. Moreover, reliability as a measure of the extent to which a variable or a set of variables is consistent in what it is intended to measure was assessed by calculating Cronbach's alpha. The closer the value comes to one, the stronger the connection between the different variables is assumed. Values greater than 0.7 are generally regarded as markers of high reliability.

## 3. Results

Of the enrolled 66 patients, 51 (77%) were female, 15 (23%) male, their mean age was 58.3 (45–83) years, with a mean disease duration of 40 (3–365) months. For the radiological assessment, the Kellgren Lawrence classification was applied. Four patients (6%) were Kellgren Lawrence stadium I, 16 (24%) in stadium II, 31 (47%) stadium III, and the remaining 15 patients (23%) in stadium IV. Patients with isolated thumb base osteoarthritis were not included into the study. Considering gender distribution, disease duration, and radiological status, the patient group can be regarded representative for the overall HOA patients being treated at our clinic. All patients were current users of either paracetamol or NSAIDs. In eleven NSAID-treated patients, the currently used drug was changed because of inefficacy at the time of their first assessment and subjects were reassessed after a mean of 39 (±4 SEM) days. All patients were Caucasian and their mother tongue was German. All of them gave written informed consent to be enrolled into the study according to the declaration of Helsinki. The design of the study has been approved by the local ethics committee.

The majority of sixty two patients (94%) properly completed both questionnaires without missing data. Four of the fifteen male patients (27%) completed M-SACRAH without any problems but had incomplete AUSCAN questionnaires (e.g., they did not fill in all physical function questions asserting never to perform these activities, i.e., closing bracelets) and were therefore excluded from further analysis.

AUSCAN pain amounted to 41.9 (±2.9 SEM), AUSCAN stiffness to 53.1 (3.7), and AUSCAN function to 42.6 (3.2). M-SACRAH-function amounted to a mean of 25.4 (2.4), M-SACRAH-stiffness to 42.6 (3.0), and M-SACRAH-pain to 43.7 (3.1). The mean total M-SACRAH was 37.2 (2.4).

The single domains of the two scores correlated significantly pain: *r* = 0.73 (*P* < .0001), stiffness *r* = 0.75 (*P* < .0001), and function *r* = 0.76 (*P* < 0.0001).

The four items which are identical in both scores also correlated significantly: *r* = 0.88 (*P* < .0001) for “pain at rest,” *r* = 0.78 (*P* < .0001) for “morning stiffness,” *r* = 0.75 (*P* < .0001) for “turning taps,” and *r* = 0.80 (*P* < .0001) for “doing up buttons.”

Gender-specific results for the AUSCAN and M-SACRAH did not differ significantly. However, it seems noteworthy that for all domains except the AUSCAN function a trend to higher values was seen in male patients, see Table 3.

Changes of both scores following a therapeutic change, for example, from an ineffective NSAID to another one, were not statistically significantly different, however, the respective changes of the mean values were well comparable for both items, see Table 4.

Mean PGA amounted to 39 (±3 SEM), mean PhGA to 23 (2). Correlations between PGA and the single domains of the AUSCAN and M-SACRAH as well as the total M-SACRAH reached statistical significance. The same, PhGA and the single domains of either instrument correlated significantly. The best correlation, however, could be demonstrated for the total M-SACRAH and PGA (*r* = 0.65; *P* < .0001), see Table 5.

Reliability testing of both scores was carried out by Cronbach's alpha, which amounted to 0.916 for the total M-SACRAH and to 0.952 for the total AUSCAN, indicating high internal consistency.

The complete results of exploratory factor analysis for the M-SACRAH items and AUSCAN items are given in Tables 6 and 7. This statistical procedure revealed both aggregate scores to be three dimensional, while the respective single-domain scores were found to be strictly one dimensional, see also the backgrounds within the respective tables.

## 4. Discussion

M-SACRAH and AUSCAN, two self-administered patient centered questionnaires, were compared in a group of HOA patients. We were able to show an equal ability of either instrument to describe physical function, pain, and stiffness in this specific patient group.

To facilitate an objective follow-up of patients suffering from HOA, the application of appropriate aggregate scores to describe the patient's status has been considered desirable aside the assessment of pain and functioning [5, 17].

The M-SACRAH was developed from its more complex predecessor, the SACRAH, which was constructed using a Delphi approach including rheumatologists and occupational therapists [13, 14]. The modified score reduced item set was reached by excluding all items of the SACRAH, which correlated with a coefficient of equal or more than 0.7. The AUSCAN in contrast was developed by first collecting a large number of items through interviews with 50 HOA patients. This item pool was rationalized according to prevalence, frequency, and importance to the patient. Subsequently, 15 “candidate” items were selected for the questionnaire. A second group of 24 “reserve” items was kept for addressing a methodological issue relating early versus late item reduction in index construction, which has not been published yet [8, 11].

As the AUSCAN is not unrestrictedly available, even for scientific purposes, a linguistic validation procedure of these items had to be performed. However, if a questionnaire is translated into another language, a linguistic validation is necessary but not sufficient unless the psychometric characteristics have been verified. Thus, the following psychometric evaluation can also be regarded as a proof of this instrument validity [18].

Both scores encompass a comparable number of questions but put a different emphasis on the investigated domains function, stiffness, and pain due to their development process. We, therefore, decided to investigate as to whether these differences would take effect on the results of the scores.

The absolute values indeed significantly differed with respect to the function and stiffness domains. The differences in the function domain can be seen due to the different scope of questions addressing functions requiring physical force like holding a pan or wringing out washcloths that are highlyer represented within the AUSCAN questionnaire. Regarding stiffness, the results of the M-SACRAH were lower than those of the AUSCAN obviously due to the presence of a second item “stiffness later in the day following inactivity” which yields significantly lower results than the item “morning stiffness” shared by both scores. Absolute values for pain in contrast to the two other domains did not differ as the items “pain at gripping,” “at lifting” and “at turning” of the AUSCAN obviously relate to “pain during hard work” of the M-SACRAH.

Despite these differences regarding absolute values, however, an expected consistently high correlation between the three domains of both instruments could be found indicating that both scores describe the investigated cohort equally well.

Internal consistency, as assessed by Cronbach's alpha, was found excellent for both the AUSCAN as well as the M-SACRAH, which indeed is caused by the high number of items. As alpha can also be regarded as a measure of redundancy, the small differences between both scores are based on the greater number of items included into the AUSCAN, as commonly reliability coefficients of compositive indices increase by an increasing number of single components [16].

The consistently high correlation between the single items of both instruments can be regarded a strong marker for convergent validity.

Discriminant validity has been tested in the SACRAH, healthy controls serving as comparative group [13]. Addressing this topic, recent data have revealed significant differences of the SACRAH as well as the M-SACRAH in HOA and RA patients [15]. Detailed data on validity and factor structure of the AUSCAN have been published in 2006 [19, 20].

Despite no significant difference between female and male patients' results, a predominance of items addressing household activities in the AUSCAN was obvious. Concordingly, we observed a considerable number of male patients (27%) who did not complete one or more questions of the function domain of the AUSCAN, some of them adding a written commentary that they would never perform such work. Although the investigated number of male patients is small, a considerable part of those considered the scope of the AUSCAN's items as not fully suitable describing their difficulties in daily life. Thus, a detailed investigation of this subject seems to be appropriate as, in addition, male patients tended to score higher in the AUSCAN as well as in the M-SACRAH. Following a change of NSAID due to inefficacy, neither the AUSCAN nor the M-SACRAH showed significant changes, obviously due to the small number of patients. Nevertheless, changes in the three domains of the two instruments were of the same magnitude and toward the same direction. The responsiveness to therapy has been shown for the AUSCAN and the SACRAH in previous publications [5, 13].

The correlations of both scores with PGA underline either ability to express the patient's present situation. Among those, the strongest correlation could be found for PGA and the total M-SACRAH, which can be regarded an advantage of this “total” aggregate score. PhGA did not correlate that strongly with both instruments. This can be seen in line with our findings about the different view of physicians and patients concerning their present disease activity as well as the respective changes in RA patients [21].

An important aspect of this study was to test the construct validity of both scores, which was done by principal component analysis. Both scores, when analyzed as an aggregate, appeared to be tridimensional instruments, reflecting the three domains covered by the scores. Although no composite AUSCAN value was proposed, it would be statistically justified to give [8]. As it is the case for the M-SACRAH, an aggregate result of the AUSCAN is supposed to measure the severity of the underlying disease [14].

As expected, this statistical approach revealed a considerable number of redundant questions. Thus, it would be possible to reduce the number of items of both scores significantly according to these results. A possible future perspective would be the validation of a short form (SF-) SACRAH or AUSCAN in order to facilitate the application of these instruments in daily routine [22].

Recent studies on the measurement of functioning in HOA patients have compared several questionnaires (HAQ, AUSCAN, Cochin scale, FIHOA, SACRAH, and AIMS2-SF) based on the International Classification of Functioning, Disability, and Health (ICF) [23]. Among those, SACRAH and AUSCAN showed the lowest diversity ratio, in contrast, AIMS2-SF the highest. The authors conclude that clinicians, when selecting an instrument for comprehensive measurement of functioning, are advised to include both one instrument with a low diversity ratio (for disease specific aspects) and another instrument with a high diversity ratio (for broader aspects of functioning including some aspects of participation).

Hand osteoarthritis and its specific assessment and treatment have received less attention than hip and knee OA in the past. Recent efforts to this entity mirror an increasing scientific interest in HOA, ending up with the creation and application of patient-centered outcome measures [2, 3].

Future research on HOA is expected to focus on good longitudinal studies and improved interventions.

In summary, apart from the observed differences in absolute values, both instruments can be regarded equally well able to describe physical function, pain, and stiffness of patients suffering from HOA. They may, therefore, both be considered equally suitable as core items in the evaluation of these patients.

## Figures and Tables

**Table 1:** Items and results of the AUSCAN. (Identical questions in both questionnaires are given in italics.)

Domain question mean (SEM)	
Pain	
Pain at rest	35.8 (3.4)
Pain at gripping	45.7 (3.8)
Pain at lifting	46.3 (3.6)
Pain at turning	45.2 (3.8)
Pain at squeezing	36.7 (3.6)

Stiffness	
Morning stiffness	53.2 (3.7)

Physical function	
Turning taps/faucets on	30.1 (3.3)
Turning doorknob/handle	24.8 (3.1)
Doing up buttons	35.0 (3.7)
Fastening jewellery	47.4 (4.2)
Opening a new jar	57.0 (4.1)
Carrying full put one hand	47.8 (4.1)
Peeling vegetables/fruit	37.4 (3.7)
Picking up large heavy objects	47.8 (4.1)
Wringing out washcloths	53.9 (4.1)

**Table 2:** Items and results of the M-SACRAH. (Identical questions in both questionnaires are given in italics.)

Domain question mean (SEM)	
Function	
Locking/unlocking of a door	21.9 (2.6)
Buttoning and unbuttoning shirt/blouse	31.1 (3.2)
Turning the water tap	28.1 (3.1)
Fastening or unfastening a zip	24.1 (3.0)
Tying shoelaces	28.4 (3.3)
Unscrewing cap of a tube of toothpaste	26.7 (2.9)
Turning the pages of a newspaper	18.9 (2.3)
Writing	24.0 (2.8)

Stiffness	
Morning stiffness	53.8 (3.6)
Stiffness later in the day following inactivity	31.3 (3.1)

Pain	
Pain during intensive work	47.7 (3.6)
Pain at times of inactivity	39.6 (3.5)

**Table 3:** Gender differences of the single domains.

	Female patients	Male patients	*P*-values
AUSCAN pain	41.1 (3.3)	45.9 (6.4)	.54
MSACRAH pain	43.3 (3.5)	45.6 (5.4)	.77
AUSCAN stiffness	52.8 (4.1)	54.4 (8.6)	.87
MSACRAH stiffness	42.4 (3.4)	43.2 (6.6)	.92
AUSCAN function	43.2 (3.6)	39.4 (6.9)	.65
MSACRAH function	24.2 (2.5)	30.9 (6.6)	.28

**Table 4:** Mean difference (SEM = standard error of the mean) of domain scores before and after NSAID change due to inefficacy.

	Pre-NSAID change	Post-NSAID change	*P*-values
AUSCAN pain	52.7 (8.9)	41.9 (7.2)	.16
MSACRAH pain	51.1 (9.5)	41.7 (6.4)	.23
AUSCAN stiffness	57.8 (9.6)	45.5 (8.3)	.19
MSACRAH stiffness	49.0 (6.6)	36.5 (6.8)	.57
AUSCAN function	42.2 (10.2)	44.9 (7.4)	.84
MSACRAH function	27.2 (6.9)	1.3 (5.7)	.52

**Table 5:** Correlations of single domains and total M-SACRAH with PGA/PhGA.

	PGA	PhGA
AUSCAN pain	*r* = 0.59 (*P* < .0001)	*r* = 0.56 (*P* < .0001)
M-SACRAH pain	*r* = 0.56 (*P* < .0001)	*r* = 0.50 (*P* < .0002)
AUSCAN stiffness	*r* = 0.49 (*P* < .0003)	*r* = 0.37 (*P* < .0059)
M-SACRAH stiffness	*r* = 0.53 (*P* < .0001)	*r* = 0.45 (*P* < .0007)
AUSCAN function	*r* = 0.55 (*P* < .0001)	*r* = 0.40 (*P* < .0025)
M-SACRAH function	*r* = 0.55 (*P* < .0001)	*r* = 0.46 (*P* < .0005)
M-SACRAH total	*r* = 0.65 (*P* < .0001)	*r* = 0.54 (*P* < .0001)

**Table 6:** Principal component analysis of the M-SACRAH items. Rotated component matrix (item loading).

Question	Component 1	Component 2	Component 3
M-SACRAH 1	0.53	0.003	0.949
M-SACRAH 2	0.602	0.511	0.41
M-SACRAH 3	0.687	0.328	0.237
M-SACRAH 4	0.785	0.246	0.002
M-SACRAH 5	0.648	0.364	0.031
M-SACRAH 6	0.686	0.383	0.328
M-SACRAH 7	0.845	−0.048	−0.022
M-SACRAH 8	0.765	−0.016	−0.024
M-SACRAH 9	0.186	0.808	0.051
M-SACRAH 10	0.201	0.790	−0.061
M-SACRAH 11	0.496	0.587	0.134
M-SACRAH 12	−0.20	0.888	0.044

Extraction method: principal component analysis.

Rotation method: varimax with Kaiser normalization.

The backgroud indicates questions loading on the same factor.

**Table 7:** Principal component analysis of the AUSCAN items. Rotated component matrix (item loading).

Question	Component 1	Component 2	Component 3
AUSCAN 1	−0.35	−0.006	−0.776
AUSCAN 2	0.169	0.902	0.159
AUSCAN 3	0.539	0.626	0.149
AUSCAN 4	0.580	0.598	−0.099
AUSCAN 5	0.259	0.744	−0.297
AUSCAN 6	0.539	0.442	0.120
AUSCAN 7	0.551	0.455	0.362
AUSCAN 8	0.898	0.109	−0.184
AUSCAN 9	0.833	0.286	0.297
AUSCAN 10	0.740	0.462	0.329
AUSCAN 11	0.675	0.499	0.309
AUSCAN 12	0.360	0.712	0.396
AUSCAN 13	0.815	0.327	−0.039
AUSCAN 14	0.439	0.699	0.059
AUSCAN 15	0.760	0.490	0.141

Extraction Method: principal component analysis.

Rotation Method: varimax with Kaiser normalization.

The background indicates questions loading on the same factor.
